# Celiac disease in type 1 diabetes mellitus

**DOI:** 10.1186/1824-7288-38-10

**Published:** 2012-03-26

**Authors:** Maria Erminia Camarca, Enza Mozzillo, Rosa Nugnes, Eugenio Zito, Mariateresa Falco, Valentina Fattorusso, Sara Mobilia, Pietro Buono, Giuliana Valerio, Riccardo Troncone, Adriana Franzese

**Affiliations:** 1 Department of Paediatrics, "Federico II" University, Naples, Italy; 2 School of Movement Sciences (DiSIST)- Parthenope University, Naples, Italy; 3 Department of Cellular and Molecular Pathology "L. Califano", "Federico II" University, Naples, Italy

**Keywords:** Diabetes, Celiac disease, Genetic background, HLA, Dietetic compliance, Glycaemic index, Gluten free diet, Quality of life

## Abstract

Celiac Disease (CD) occurs in patients with Type 1 Diabetes (T1D) ranging the prevalence of 4.4-11.1% versus 0.5% of the general population. The mechanism of association of these two diseases involves a shared genetic background: HLA genotype DR3-DQ2 and DR4-DQ8 are strongly associated with T1D, DR3-DQ2 with CD. The classical severe presentation of CD rarely occurs in T1D patients, but more often patients have few/mild symptoms of CD or are completely asymptomatic (silent CD). In fact diagnosis of CD is regularly performed by means of the screening in T1D patients. The effects of gluten-free diet (GFD) on the growth and T1D metabolic control in CD/T1D patient are controversial. Regarding of the GFD composition, there is a debate on the higher glycaemic index of gluten-free foods respect to gluten-containing foods; furthermore GFD could be poorer of fibers and richer of fat. The adherence to GFD by children with CD-T1D has been reported generally below 50%, lower respect to the 73% of CD patients, a lower compliance being more frequent among asymptomatic patients. The more severe problems of GFD adherence usually occur during adolescence when in GFD non compliant subjects the lowest quality of life is reported. A psychological and educational support should be provided for these patients.

## Introduction

Type 1 Diabetes Mellitus (T1D) is frequently associated to other autoimmune conditions. These conditions can severely affect clinical management of the disease, especially in paediatric age.

The most frequent are autoimmune thyroid disease (AIT), celiac disease (CD), Addison's disease (AD) and vitiligo. These diseases are associated with organ-specific autoantibodies: AIT with thyroid peroxidase (TPO) and thyroglobulin autoantibodies (TG), CD with endomysial (EMA) and transglutaminase (TTG) autoantibodies, and AD with adrenal autoantibodies. Using these autoantibodies, organ-specific autoimmunity may be often detected before the development of clinical disease, in order to prevent significant morbidity related to unrecognized disease [1]. These diseases are very often clustered in the same individual and a shared genetic background probably explains this association [2].

## Genetics

The majority of autoimmune endocrine diseases, including T1D, are inherited as complex genetic traits. Multiple genetic and environmental factors interact with each other to confer susceptibility to these disorders. Genetic risk factors associated with T1D, ATD, CD and AD include HLA genes and non-HLA genes.

HLA DR4 and DR3 are strongly associated with T1D and approximately 30-50% of patients are DR3/DR4 heterozygotes. The DR3/DR4 genotype confers the highest diabetes risk with a synergic mode of action, followed by DR4 and DR3 homozygosity, respectively. The HLA-DQ (particularly DQ 2 and DQ8) locus has been found to be the most important determinant of diabetes susceptibility. Approximately 90% of individuals with T1D have either DQ2 or DQ8, compared to 40% of the general population [3]. So, the highest-risk human leukocyte antigen (HLA) genotype for T1D is DR3-DQ2, DR4-DQ8.

DR3-DQ2 shows a strong association with CD; homozygosity for DR3-DQ2 in a population with T1D carries a 33% risk for the presence of TTG autoantibodies [4].

Non-HLA genes are also involved in the predisposition to T1D and other autoimmune diseases, such as MIC-A, PTPN22, CTLA-4 [1].

## Epidemiology

Traditional studies, both in children and adults, have shown that CD occurs in patients with T1D with a prevalence that varies from 4.4 to 11.1% compared with 0.5% of the general population (Table 1 for references from) [5-14]. The mean age at diagnosis of classical CD is commonly around 2-3 years, while the mean age at diagnosis of T1D is 7-8 years. The age at onset of T1D is younger in patients with the double disease than in those with only T1D [15]. The risk of CD is negatively and independently associated with age at onset of diabetes, with an higher risk being seen in children age < 4 years than in those age > 9 years [16]. In patients with T1D, diabetes is usually diagnosed first, CD precedes diabetes onset only in 10-25% [16,17], while generally CD diagnosis in T1D patients occurs, trough the screening performed at diabetes onset, in 70-80% of patients with a median age > 8 years. Some authors hypothized that in genetically susceptible patients one disease could predispose to another. Particularly, it has been suggested that untreated (latent or silent) CD could be an immunological trigger and induce diabetes and/or thyroid disorders due to gluten as a driving antigen [18]. In accordance with this, the prevalence of autoimmune disorders in CD is closely related to age at diagnosis or, in other words, to the duration of exposure to gluten [19] and thyroid-related antibodies tend to disappear during twelve months of gluten-free diet, like CD-related antibodies [20]. However, at present, it is unknown whether treatment of CD reduces the likelihood of developing autoimmune disorders, or changes their natural history and actually others found no correlation between duration of gluten exposure in adult CD and risk of autoimmune disorders [21].

**Table 1:** Prevalence of CD in patients with T1D in recent literature (2004-2011)

Reference	Country	**N**.	Age (yr)	Screening	Prevalence (%)
Cerutti et al. 2004	Italy	4322	11.8 ± 4.2	AGA + EMA	6.8

Contreas et al. 2004	North Italy	357	Children	EMA	7

Sanchez et al. 2005	Germany	281	Children	AGA + EMA	6.4

Araujo et al. 2006	Brasil	354	Children	TG	10.5

Goh et al. 2007	UK	113	Children	EMA + TG + AGA	4.4

Larsson et al. 2008	Sweden	300	< 20	EMA	10

Karavanaki et al. 2009	Greece	144	12.3 ± 4.6	TG	4.8

Djuric et al. 2010	Serbia	121	Mean 10.8	TG	5.79

Bhadada et al. 2011	India	189	10.81 ± 7.3	TG	11.1

Gabriel S et al. 2011	Romania	119	11 ± 4	TG	9.2

## CD clinical symptoms

The most severe CD-related symptoms are generally related to gastrointestinal malabsorption and include malnutrition, failure to thrive, diarrhea, anorexia, constipation, vomiting, abdominal distension, and pain. These features are more common in children younger than three years of age. Non-gastrointestinal symptoms of CD include short stature, pubertal delay, fatigue, vitamin deficiencies, and iron deficiency anemia and are more commonly observed in older children. The gastrointestinal presentation of CD rarely occurs in T1D patients (< 10%), but many patients with CD and T1D are either asymptomatic (silent CD) or present only mild symptoms [17,20,22]. Furthermore, the wide spectrum of CD include also subjects with positive celiac-related antibodies without diagnostic small-bowel mucosal villous atrophy. This condition is defined as potential celiac disease (pot-CD) [23-25]. Data from the majority of childhood diabetes care centers in Italy showed that prevalence of pot-CD patients in this population (higher in females than males) is 12.2%, while the prevalence of pot-CD in the CD control population is 8.4% and only few of them present CD-related symptoms [26].

## CD-screening

Diagnosis of CD is regularly due to screening protocols which are widely recommended and performed. Actually diagnosis is commonly performed by means of TTG IgA (confirmed by EMA) or TTG-IgG if IgA-deficiency is present. Screening has to performed at followed times: 1) at the time of diabetes onset, 2) yearly in the first 4 years of follow up, 3) each 2 years in the successive 6 years of follow up [27,28]. In the presence of CD-related antibodies positivity it is mandatory to perform bowel biopsy to confirm diagnosis of CD, even if in very recent guide-lines of ESPGHAN Society [29] it is proposed that in evident CD-cases it is possible to avoid biopsy (4 main criteria).

## CD-treatment

GFD should be proposed actually only in patients with mucosal atrophy.

In patients with overt CD, identifying and treating CD with gluten free diet (GFD) surely confer benefit in reducing/resolving malabsorption, infertility, osteoporosis, poor nutrition, impaired growth and long-term malignancy risks and mortality rates [30-32]. Similarly, children with T1D and symptomatic CD benefit from GFD [33] and also metabolic control of diabetes could be ameliorated [34].

On the contrary, in symptom-free patients weight gain and bone mineral density (BMD) changes have been non-univocally described as benefit [35-37]. The different viewpoints highlight the need of a prolonged follow up in patients affected by T1D and asymptomatic CD to clarify the role of GFD. Some authors argument that GFD in asymptomatic CD-T1D patients should be opportunely proposed but not excessively stressed [38,39].

Finally, no definite consensus exists among experts about to treat by GFD pot-CD patients, in whom recently it has been suggested that GFD could be a benefit [40]. Concerning to the natural history of patients whit pot-CD, a recent study shows that 30% of these patients develops overt CD in a three years follow-up and underlines the necessity of re-testing [41]. However no data are available about the follow-up of patients with T1D and pot-CD.

Surprisingly, intestinal inflammation has been described also in T1D patients without CD-related antibodies and structurally normal intestinal mucosa [42]. According to this, our group has observed a gluten-related inflammation either in rectal either in small bowel mucosa of children with T1D [43,44]. It can be speculated that gluten could be an optimal candidate to stimulate an abnormal innate immune reaction in intestinal mucosa due to its pro-inflammatory characteristics. It remains a crucial issue to establish if the extended intestinal inflammation in T1D is gluten-dependent and whether it precedes the occurrence of the disease.

## Bone impairment: a hidden threat

In patients with only T1D it is possible to demonstrate impairment of bone metabolism and structure, specially in relationship with duration and/or poor control of diabetes [45]. Furthermore CD also have been underlined as cause of bone impairment. Clinical observation indicates that clustering of three autoimmune diseases (T1D, CD and generally thyroiditis) significantly increases the occurrence of osteopenia (37.5%). It is possible that bone impairment might be considered not only a complication due to endocrine or nutritional mechanisms, but also a consequence of an immunoregulatory imbalance [46]. In fact osteoclasts are now considered as the innate immune cells in the bone, since they are able to produce and respond to cytokines and chemokines. Bone remodelling involves complex interactions between osteoclasts and other cells in bone microenvironment (marrow stromal cells, osteoblasts, macrophages, T-lymphocytes and marrow cells) [47]. Several cytokines, like the cytokine receptor activator of NFkB ligand (RANKL) and the macrophage colony stimulating factor (M-CSF), can promote osteoclast formation and activity. Also osteoprotegerin (OPG), a circulating secretory glycoprotein, could have a role in bone remodelling in children with T1D because it could promote differentiation, fusion, survival, activation and apoptosis of the osteoblasts. Alterations or abnormalities of the RANKL/OPG system have been implicated in different metabolic bone diseases characterized by increased osteoclast differentiation and activation, and by enhanced bone resorption [48].

In patients affected by both T1D and CD, the risk of developing osteopenia is also influenced by the compliance to GFD. In fact, osteopenia occurs more frequently in patients with diabetes and CD with poor compliance to GFD [49]. Recent observations indeed indicated an imbalance of cytokines relevant to bone metabolism in untreated celiac patients' sera and the direct effect of these sera on in vitro bone cell activity. In particular the RANKL/osteoprotegerin (OPG) ratio was increased in patients not on gluten-free diet [46].

In conclusion osteopenia seems to be a new occult problem in CD patients, in T1D patients and in patients with two or three immunological diseases, depending also on GFD.

## GFD composition

Diet is a fundamental part of the treatment in both T1D and CD. However GFD composition could present some problems for diabetic people (Table 2).

**Table 2:** Variations of HbA1c, BMI gain and height velocity after GFD in children with T1D-CD

Reference	HbA1c	BMI gain	Heigh velocity
Westman et al.	unchanged	Unchanged	unchanged

Saukkonen et al.	unchanged	Increased	unchanged

Amin et al.	reduced	Increased	unchanged

Saadah et al.	unchanged	Increased	unchanged

Valletta et al.	unchanged	Unchanged	unchanged

The glycemic index (GI) provides an indirect measure of the ability of a food to raise blood glucose.GI is retained a direct index of absorption of carbohydrates, being: "the area under curve of blood glucose after eating a food containing a determined quantity of carbohydrate". White bread (GI = 100) is usually compared as reference value. In normal subjects ingestion of foods with high GI leads to a rapid blood glucose increase causing a marked insulin response. In diabetes, diet containing food with high GI is considered inopportune because in condition of insulin deficiency (T1D) or insulin inefficacy (type 2 diabetes) the normal insulin response is not obtainable; traditionally the common diet of the diabetes people consists principally in foods with low GI. In 2002 American Society for Clinical Nutrition published an international table, revised by an older published of the sixties, which shows the GI of most common foods, evaluated in comparison to glucose and to white bread [50], (Table 3) where gluten-free products have higher GI-foods than similar products gluten containing. In Paker's study [51] six types of gluten-free foods are compared with white bread containing gluten. These foods were eaten from 11 adult patients with type 2 diabetes and blood glucose was measured after eating. Results showed no difference about GI among gluten-free foods and those containing gluten. On the contrary, Berti et al. show a higher blood glucose curve for gluten-free foods, although with similar insulin curves and with contradictory results between in vivo and in vitro analysis. (Table 4), [52]). Specific studies both in healthy patients and in type 1 and type 2 diabetic patients should be necessary, particularly in pediatric age.

**Table 3:** GI of some gluten-free foods, compared to glucose and white bread

	GI glucose = 100	GI bread = 100
Gluten-free multigrain bread	79 ± 13	113

Gluten-free white bread	76 ± 5	108 ± 7

Gluten-free fiber enriched bread	73 ± 4	104 ± 5

**Table 4:** GI of gluten-free foods evaluated in vivo, compared to white bread (= 100)

Food	GI
Gluten-free bread	230

Gluten-free pasta	255

Quinoa	186

In addition, gluten-free foods are prepared using corn flour, rice and wheat, where the percentage of fiber, carbohydrate, fats and micronutrients isn't completely known. Scarce contrasting data generally describe in gluten free foods composition few proteins, more fat and few fibers than gluten containing foods. (from SCHAR website and Ministry of Agriculture website, Tables 5 and 6). In addition in the review of Kupper [53] GFD seems to can be the cause of a multiple nutrients deficiency, especially of vitamin B, vitamin D, calcium, magnesium, iron, zinc, but sources of his information are not well documented. Finally Berti et al. [52] reported higher amount of fats in gluten-free bread then those with gluten, but the same amount of fibers (Table 7).

**Table 5:** Nutritional composition of gluten free and containing gluten foods

Products	PROT (g)	CHO (g)	SUGARS (g)	FATS (g)	FIBER (g)	KCALS
Flour 00	11	77.3	1.7	0.7	2.2	340

Gluten-free flour	1.2	86.3	1.5	0.8	4.5	357

Crackers containing gluten	9.4	80.1	2.5	10	2.8	428

Gluten-free crackers	5.2	74.7	5.2	12.7	2.9	434

Cereal flakes containing gluten	6.6	87.4	10.4	0.8	3.8	361

Gluten-free cereal flakes	8	80	5.3	16	5	361

Rusks containing gluten	11.3	82.3	2.2	8.2	3.5	408

Gluten-free rusks	4.8	82.9	5.4	1.4	3.6	425

Pasta containing gluten	10.9	79.1	4.2	2.5	2.7	353

Gluten-free pasta	9	73.7	0.4	7.9	2.2	353

Biscuits	6.6	84.8	18.5	14.3	2.6	416

Gluten-free biscuits	2.7	79.9	16.1		0.8	459

**Table 6:** Portion size, macronutrient and micronutrient composition of test meal.

	White bread	GF biscuits	GF white Unsliced bread	GF fibre Sliced bread	GF white Sliced bread	GF fibre Unsliced bread	GF pasta
Serving (g)	107	73	101	119	101	119	64

Energy (kcals)	232	335	221	225	221	236	230

Protein (g)	8.1	2.55	3.03	3.57	3.03	3.57	5.05

Carbohydrate (g)	50	50	50	50	50	50	50

(g sugars)	3.21	17.5	4.54	5.36	4.50	5.35	0.61

Fat (g)	1.39	13.87	1.01	1.19	1.01	2.38	1.02

(g satured)	0.32	4.38	0.50	0.60	0.50	1.19	0.32

Fibre (g)	1.61	2.92	1.01	5.95	1.01	7.7	0.96

Sodium (g)	0.56	0.37	0.51	0.47	0.51	0.35	0.05

GF = gluten-free

**Table 7:** Weight of meal and nutrient composition of 50 g available carbohydrate portions of the foods studied as served.

Tests foods	Weight of meal (g)	Protein (g)	Water (g)	Carbohydrate (g)	Fat (g)	Total dietary fibers (g)
Bread	100	9.4	31.4	50	3.6	2.8

GF Bread	125	5.7	38.5	50	9.7	3.9

Gf Pasta	156	3.2	61.2	50	0.6	2.1

Quinoa	320	3.4	75.9	50	2.0	2.8

GF = gluten-free

## Compliance/adherence to GFD and quality of life (QOL)

Adherence to GFD among T1D-CD patients, in our experience, is generally good in patients who experienced clear clinical symptoms of CD, but is scarce among patients with few symptoms or asymptomatic. In Table 8 a summary of the data of literature is presented, but authors did not specify whether patients had experienced symptoms; data of our group are also presented [36]. In contrast with T1D population, dietary compliance in CD patients (without T1D), seems to be higher: approximately 73% of patients followed the diet strictly [54]. Probably for a patient with T1D, already engaged in coping day by day a complex chronic disease, the addition of a second "limiting" condition, is a remarkable discomfort [55]. Consequently, in the case of double diagnosis (T1D + CD), it is very difficult to manage patients who did not experienced CD symptoms.

**Table 8:** Adherence to the GFD in children with T1D-CD

Reference	Country	Prevalence (%)
Acerini et al.	United Kingdom	Partial

Westman et al.	Australia	30

Valerio et al.	Italy	59

Saadah et al.	Australia	25

Studies on the compliance/adherence to GFD in non diabetic people showed that, in relation to the social life, children usually have a better compliance to GFD than adults [56]. In a follow-up of 10 years in the Netherlands conducted on children from 2 to 4 years who received CD diagnosis by screening, authors described a general improvement of health without deterioration in QOL [57]. In concordance Kolsteren showed that the QOL of celiac children is quite similar to that of other children [58]. Usually the difficulty with the diet occurs when the patient became adolescent, because she/he needs to feel equal to peers, especially when she/he decides to go out to eat and more acutely she/he feels limits imposed by GFD. According to Wagner et al. [59] celiac adolescents non compliant with GFD reported a lower general QOL, more physical problems, a higher burden of illness, more family troubles, and more problems in leisure time than adolescents who are compliant with GFD. No differences between compliant patients with CD and adolescents without any chronic condition were found in all QOL aspects.

It is also important remark that the balance between GFD adherence and daily life is difficult to achieve for the child/adolescent who is also affected by an other chronic disease such as T1D. The need to coordinate insulin therapy with proper nutrition and a healthy lifestyle, in order to maintain adequate metabolic control, is already a considerable effort for the young T1D-patient and families [60]. Rebellions are frequent especially in adolescents, who are already feeling diabetes as very "invasive" for all the aspects of daily life and who receive a further "restriction" constituted by the GFD. Consequently there it could be the risk, especially if this proposal is not properly, to elicit a response of complete rebellion which will endanger not only the adherence to the GFD [36,54,55], but also the entire management of T1D, causing a sharp deterioration of general compliance and increasing the risk of severe acute complications (recurrent ketoacidosis, unawareness hypoglycemia). In addition it is possible to think that this further limit could be a trigger also of eating disorders in adolescent patients, being eating disorders not rare and previously reported in diabetes. Regarding QOL, Sud et al. [61] in children with T1D-CD showed that the double diagnosis appears to have a minimal impact on QOL, even if patients' parents reported a very important difficulty on management. It is interesting that not significant differences in QOL were observed with regard to age at CD diagnosis and duration, or on the basis of adherence with a GFD. Furthermore parents of T1D-CD children did express greater concern about their child's social functioning.

## Conclusions

Prevalence of CD among children with T1D is significantly higher than in non diabetic children. In a large proportion CD is asymptomatic or characterized by modest/atypical symptoms. Periodic screening of CD auto-antibodies is mandatory. CD diagnosis requires the biopsy confirmation and it is necessary to prescribe GFD in the presence of mucosa impairment. Concerning the clinical benefits of GFD in T1D-CD patients, data are contrasting, except in severely symptomatic patients.

Osteopenia seems to be a new occult problem in CD patients, in T1D patients and in patients with two or three immunological diseases, depending also on GFD.

It is unclear whether GFD composition could present any disadvantages regarding of glycemic index, fibers, percentage of fat and micro-nutrients. Data are not univocal on this point.

Communication of the need of GFD in patients with T1D-CD is particularly delicate, especially in adolescents where it is possible to trigger rebellion behaviors. The coexistence of these two diseases in the same patient requires care by clinicians and probably a specific psychological approach.

## Abbreviations

AIT: Autoimmune thyroid disease; AD: Addison's disease; BMD: Bone mineral density; CD: Celiac disease; EMA: Endomysial autoantibodies; GFD: Gluten-free diet; HbA1c: Glycated hemoglobin; HLA: Human leukocyte antigen; pot-CD: Potential celiac disease; QOL: Quality of life; T1D: Type 1 diabetes; TG: Thyroglobulin autoantibodies; TPO: Peroxidase autoantibodies; TTG: Transglutaminase autoantibodies

## Competing interests

The authors declare that they have no competing interests.

## Authors' contributions

MEC (MD), EM (MD), PB (MD): have been involved in drafting the manuscript, except "Composition diet", "Compliance/adherence to GFD and quality of life" and "Genetics". EZ (Psy. D): has been involved in drafting "Compliance/adherence to GFD and quality of life". MF (MD), VF(MD): acquisition of data. SM (Dietitian): has been involved in drafting "Composition diet". RN (MD): has been involved in drafting "Genetics". GV (MD), RT (MD): have rivisited critically the manuscript. AF (MD): conception and design of the manuscript
